# Volatilomics Reveals Potential Biomarkers for Identification of Renal Cell Carcinoma: An In Vitro Approach

**DOI:** 10.3390/metabo10050174

**Published:** 2020-04-27

**Authors:** Filipa Amaro, Joana Pinto, Sílvia Rocha, Ana Margarida Araújo, Vera Miranda-Gonçalves, Carmen Jerónimo, Rui Henrique, Maria de Lourdes Bastos, Márcia Carvalho, Paula Guedes de Pinho

**Affiliations:** 1 UCIBIO, REQUIMTE, Laboratory of Toxicology, Faculty of Pharmacy, University of Porto, 4050-313 Porto, Portugal; silviagfrocha@gmail.com (S.R.); ana.margarida.c.araujo@gmail.com (A.M.A.); mlbastos@ff.up.pt (M.d.L.B.); pguedes@ff.up.pt (P.G.d.P.); 2 Master in Oncology, Institute of Biomedical Sciences Abel Salazar–University of Porto (ICBAS-UP), 4050-313 Porto, Portugal; 3 Cancer Biology & Epigenetics Group, Research Centre (CI-IPOP) Portuguese Oncology Institute of Porto (IPO Porto), 4200-072 Porto, Portugal; vera.miranda.goncalves@ipoporto.min-saude.pt (V.M.-G.); carmenjeronimo@ipoporto.min-saude.pt (C.J.); rmhenrique@icbas.up.pt (R.H.); 4 Department of Pathology and Molecular Immunology-Biomedical Sciences Institute (ICBAS), University of Porto, 4050-313 Porto, Portugal; 5 Department of Pathology, Portuguese Oncology Institute of Porto (IPO Porto), 4200-072 Porto, Portugal; 6 UFP Energy, Environment and Health Research Unit (FP-ENAS), University Fernando Pessoa, 349, 4249-004 Porto, Portugal

**Keywords:** renal cell carcinoma, cell lines, metabolomics, volatile compounds, HS-SPME/GC–MS, biomarkers

## Abstract

The identification of noninvasive biomarkers able to detect renal cell carcinoma (RCC) at an early stage remains an unmet clinical need. The recognition that altered metabolism is a core hallmark of cancer boosted metabolomic studies focused in the search for cancer biomarkers. The present work aims to evaluate the performance of the volatile metabolites present in the extracellular medium to discriminate RCC cell lines with distinct histological subtypes (clear cell and papillary) and metastatic potential from non-tumorigenic renal cells. Hence, volatile organic compounds (VOCs) and volatile carbonyl compounds (VCCs) were extracted by headspace solid-phase microextraction (HS-SPME) and analyzed by gas chromatography–mass spectrometry (GC–MS). Multivariate and univariate analysis unveiled a panel of metabolites responsible for the separation between groups, mostly belonging to ketones, alcohols, alkanes and aldehydes classes. Some metabolites were found similarly altered for all RCC cell lines compared to non-tumorigenic cells, namely 2-ethylhexanol, tetradecane, formaldehyde, acetone (increased) and cyclohexanone and acetaldehyde (decreased). Furthermore, significantly altered levels of cyclohexanol, decanal, decane, dodecane and 4-methylbenzaldehyde were observed in all metastatic RCC cell lines when compared with the non-metastatic ones. Moreover, some alterations in the volatile composition were also observed between RCC histological subtypes. Overall, our results demonstrate the potential of volatile profiling for identification of noninvasive candidate biomarkers for early RCC diagnosis.

## 1. Introduction

Renal cell carcinoma (RCC) accounts for more than 90% of all kidney malignancies [1] and represents the second most lethal urological cancer [2]. Comprising a heterogeneous group of tumors, RCC can be subdivided into several histological subtypes according to distinct clinical and histological features and different outcomes [3,4]. The main groups include clear cell renal cell carcinoma (ccRCC), papillary renal cell carcinoma (pRCC) and chromophobe renal cell carcinoma (chRCC), among other rare types [3]. ccRCC is the most prevalent histological subtype, representing more than 75% of all RCCs [5], followed by pRCC, which represents 10%–15% of cases [6].

RCC has a favorable prognosis when detected at an early stage [7]. However, this disease prevails asymptomatically until the advanced stages [8] and no satisfactory biomarkers for clinical management are currently used [9,10]. Despite the use of imaging techniques, such as the computed tomography scan, ultrasound exam and/or magnetic resonance, it is indispensable to perform a biopsy of the tumor to obtain a precise diagnosis [11,12]. Hence, the development of accurate and non-invasive diagnostic methods based on detection of specific biomarkers in an early stage is of upmost relevance for RCC prognosis and follow-up [9,10].

The use of metabolomics for investigation of novel biomarkers in oncobiological studies has been rising over the years [13]. Metabolomics, defined as the study of endogenously produced low molecular weight compounds, enables the identification of alterations on metabolite levels that reflect the biological activity of cancer cells [14]. RCC has been considered a metabolic disease [15] due to several alterations in metabolites associated with energy metabolism, particularly those involved in pathways for sustaining cell proliferation [10,16]. The majority of RCC metabolomic studies aims to discriminate RCC patients from controls with diagnostic purposes, but also to investigate the metabolic deviations associated with the onset of RCC, through the use of mass-spectrometry (MS) and nuclear magnetic resonance spectroscopy (NMR) [8,10,17,18,19]. These studies have found increased levels of several types of carnitines and metabolites from fatty acid metabolism in tissue and urine from RCC patients compared to control individuals [17]. The levels of carbohydrate-related metabolites as lactate, pyruvate, glutamate and glucose, among others, have also been found altered in serum/plasma [19], renal tissue [8,18] and urine from RCC patients [19]. Among all biological matrices used in metabolomic studies, urine may be considered the preferential matrix for RCC biomarker discovery since it can offer a non-invasive tool for RCC diagnosis allied with the enormous advantage to be in direct contact with the kidney. However, urine also reflects the metabolic alterations associated factors not related with the disease state, usually called confounding factors, such as age, diet, gender and medication, among others, thus introducing “metabolomic variability” and/or masking valid information [16]. To overcome this issue, we propose herein a bottom-up metabolomic approach (matrices of increasing complexity) focused on the investigation of potential RCC biomarkers in in vitro models to be confirmed and validated in urine from RCC patients in the future.

Volatile organic compounds (VOCs) are end products of cellular metabolism excreted by cells [20,21] and the analysis of those compounds in the exometabolome can provide the metabolic signature directly originated from RCC cells. Scientific evidence has demonstrated that the alterations in the volatile part of exometabolome (VOCs and volatile carbonyl compounds (VCCs) profiles) reflect the genetic mutations, adaptations and modifications in biochemical pathways of tumor cells [21]. Therefore, these compounds can be used as potential biomarkers for RCC detection and diagnosis. The use of in vitro models to study the volatile signatures of cancer cells, thus avoiding the bias introduced by confounding factors, has already been reported by other authors [21,22,23,24,25]. Indeed, deviations in the levels of VOCs have been proven for prostate [21], lung [22,23], bladder [24] and breast [25] cancer cells, whereas the dysregulations occurring in the volatile exometabolome profile of RCC cells are still unexplored. The volatile signature of RCC has only been investigated using more complex matrices, such as urine, for which significant differences in VOC levels have been found between patients and control individuals [26,27].

Hence, this study aimed to investigate, for the first time, the performance of VOCs and VCCs in discriminating RCC cell lines (769-P, 786-O, Caki-1, Caki-2 and ACHN) from one non-tumorigenic renal cell line (HK-2). We further investigated which metabolites might discriminate RCC cell lines according to their histological subtype (ccRCC vs. pRCC) and metastatic potential (metastatic vs. non-metastatic RCC). Prior to analysis by gas chromatography-mass spectrometry (GC–MS), VOCs present in the extracellular medium were directly extracted by headspace solid-phase microextraction (HS-SPME), while VCCs were first derivatized before HS-SPME to enhance the detection of aldehydes (CHO-) and ketones (CO-) [28]. Knowledge on deviations occurring in the levels of volatiles in RCC cells may provide new and specific biomarker candidates to be validated in urines from RCC patients in the future and, therefore, to obtain a powerful tool enabling faster non-invasive RCC diagnosis.

## 2. Results

**Figure 1:**
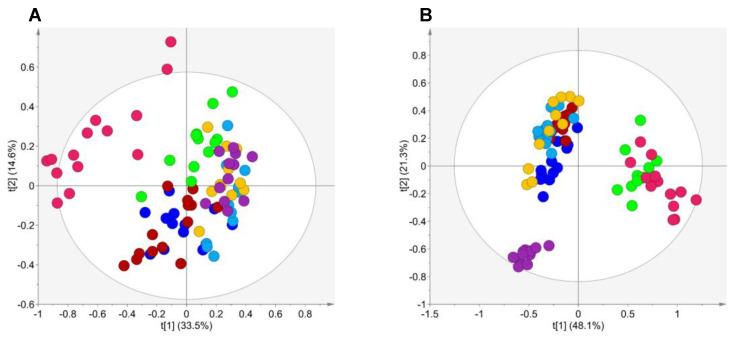
Principal component analysis (PCA) scores scatter plots obtained for the headspace solid-phase microextraction (HS-SPME)/gas chromatography–mass spectrometry (GC–MS) chromatogram data of (**A**) volatile organic compounds (VOCs) and (**B**) volatile carbonyl compounds (VCCs) of culture media of HK-2 (green, *n* = 12), 769-P (dark blue, *n* = 13), 786-O (red, *n* = 14), Caki-1 (orange, *n* = 11), Caki-2 (purple, *n* = 12), ACHN (light blue, *n* = 12) and blanks (pink, *n* = 14).

In this study, the exometabolome volatile profile of several RCC cell lines (769-P, 786-O, Caki-1, Caki-2 and ACHN) and one non-tumor renal cell line (HK-2) was analyzed to investigate whether volatile compounds might be able to discriminate tumorigenic from non-tumorigenic cells. The two most common histological subtypes, ccRCC and pRCC, at different disease stages were selected for this study, thus extending our investigation to the metabolic differences that characterize the histological subtype and tumor stage. The number of peaks detected in the extracellular medium in VOCs and VCCs analyses were 105 and 111, respectively (Figure S1). A very good reproducibility was achieved for both analytical methodologies, since all QCs were clustered in the unsupervised analysis (Figure S2). For VOCs, the principal component analysis (PCA) model including all cell lines and blanks (Figure 1A) unveiled one main cluster including all cell lines and another cluster of blanks (culture medium in the same conditions but without cells). For VCCs, three different clusters were observed in PCA (Figure 1B), namely one including the non-tumor cell line (HK-2) and blanks, a second including the cancer cell lines, and the last composed only by Caki-2. These results suggested that the RCC cell lines exhibited a distinct volatile profile when compared with the non-tumor cell line and blanks.

### 2.1. Volatile Exometabolome Signature of RCC Cell Lines versus the Non-Tumorigenic Cell Line

**Figure 2:**
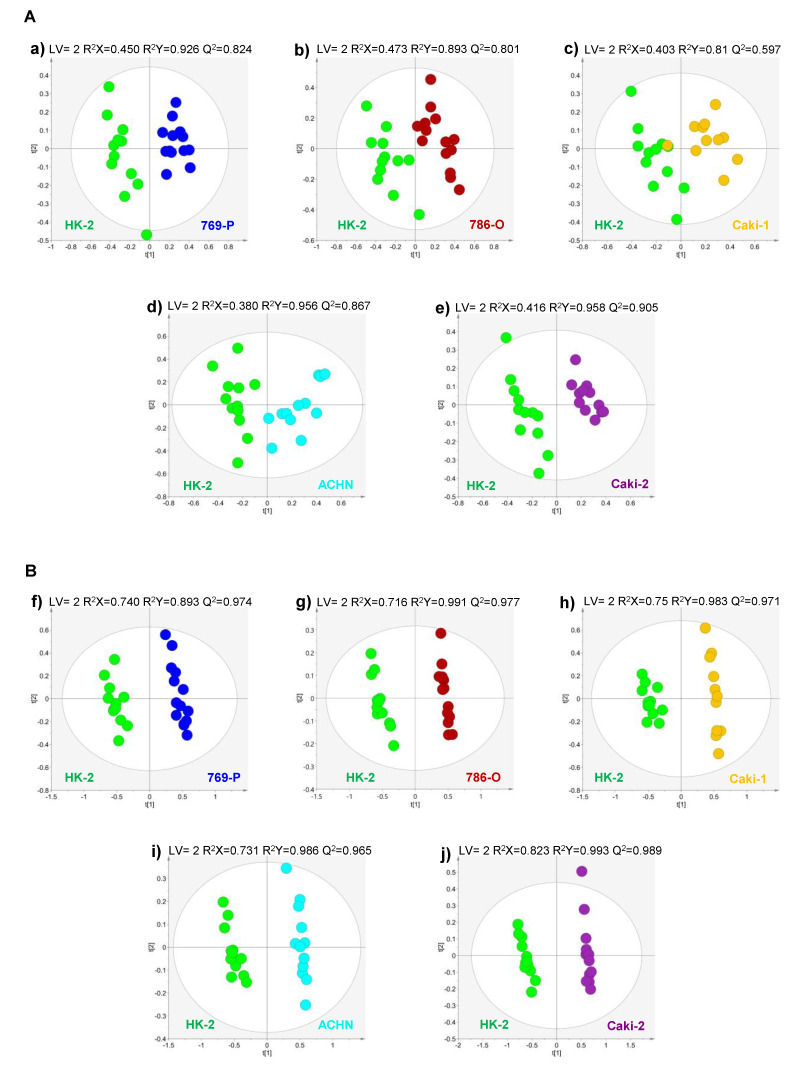
Pairwise partial least squares-discriminant analysis (PLS-DA) scores scatter plots obtained for the HS-SPME/GC–MS chromatogram data of (**A**) VOCs and (**B**) VCCs of culture media of each tumor cell line vs. the non-tumor cell line. (**a**,**f**) 769-P (dark blue, *n* = 13) vs. HK-2 (green, *n* = 12), (**b**,**g**) 786-O (red, *n* = 14) vs. HK-2 (green, *n* = 12), (**c**,**h**) Caki-1 (yellow, *n* = 11) vs. HK-2 (green, *n* = 12), (**d**,**i**) Caki-2 (purple, *n* = 12) vs. HK-2 (green, *n* = 12) and (**e**,**j**) ACHN (light blue, *n* = 12) vs. HK-2 (green, *n* = 12).

Pairwise multivariate supervised analysis (PLS-DA) was performed between each RCC cell line and the non-tumorigenic cell line for both analytical methodologies, VOCs and VCCs (Figure 2A,B, respectively). The models showed a very robust discrimination for all comparisons with high predictive ability (Q^2^ > 0.5). 

The compounds altered between each tumor cell line versus the non-tumor are shown in Table 1 and Table 2. Overall, the compound classes found altered comprised several alcohols, alkanes, alkenes, aldehydes, benzene derivatives and ketones. Interestingly, a higher number of discriminant metabolites was found for ccRCC vs. HK-2 (26 compounds; Table 1) when compared with pRCC vs. HK-2 (15 compounds; Table 2). Some alterations were found in common for all ccRCC and pRCC cell lines compared with HK-2, including increased levels of 2-ethylhexanol, tetradecane, formaldehyde, acetone and an unknown compound (Un RT 10.18, *m/z* 58), and the decrease in the levels of acetaldehyde and cyclohexanone. These results suggest the existence of a common regulatory mechanism in ccRCC and pRCC cells.

**Figure 3:**
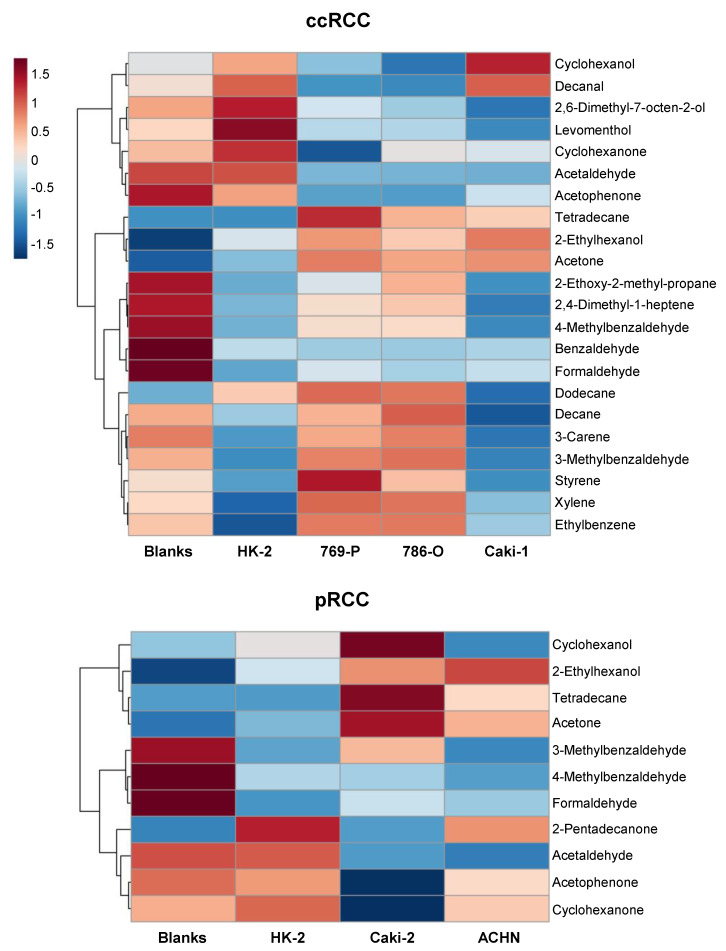
Heatmaps illustrating the mean levels (normalized peak areas) of metabolites changing in ccRCC and pRCC cell lines when compared with the non-tumor cell line (HK-2). The levels of those compounds in blanks (culture medium without cells) are also represented to aid in the interpretation of the results in terms of excretion and consumption. Rows correspond to the mean normalized peak area of each metabolite, while the columns represent each sample group.

The analysis of the volatile profile of blanks enabled the interpretation of dysregulations occurring in RCC cells in terms of excretion or consumption of culture medium nutrients. The heatmap depicted in Figure 3 shows the average peak area of each altered compound in blanks, HK-2, ccRCC and pRCC cell lines. The hierarchical clustering shown for compounds helps to interpret the patterns of compound concentrations that changed across different groups. Hence, cyclohexanol, decanal, 2,6-dimethyl-7-octen-2-ol, levomenthol, cyclohexanone, acetaldehyde and acetophenone showed a trend for lower levels in ccRCC cell lines compared with HK-2 and blanks, indicating the occurrence of an increased consumption by these cell lines, except for cyclohexanol and decanal in Caki-1 for which the levels were similar to that of HK-2 and blanks. Tetradecane, 2-ethylhexanol and acetone unveiled a tendency for higher levels in ccRCC cell lines compared with HK-2 and blanks, suggesting an increased excretion of those compounds by ccRCC cell lines. 2-Ethoxy-2-methylpropane, 2,4-dimethyl-1-heptene and 4-methylbenzaldehyde were consumed by all cell lines but at a lower extent in 769-P and 786-O compared with HK-2 (Table 1). Benzaldehyde and formaldehyde showed a tendency for consumption in ccRCC and HK-2 cell lines, but a different profile was observed for both metabolites with formaldehyde showing increased levels in ccRCC lines in relation to HK-2, while for benzaldehyde only 786-O unveiled statistically significant results when compared with HK-2 (Table 1). Decane, 3-carene, 3-methylbenzaldehyde, styrene, xylene and ethylbenzene showed a distinct profile in HK-2 and Caki-1, which seem to consume higher levels of those compounds (Table 1 and Figure 3). Regarding the pRCC cell lines, Caki-2 and ACHN showed a larger excretion of 2-ethylhexanol, tetradecane and acetone, whereas only Caki-2 unveiled a higher excretion of cyclohexanol (Table 2 and Figure 3). 3-Methylbenzaldehyde and 4-methylbenzaldehyde showed a different variation pattern for Caki-2 and ACHN (Figure 3). Formaldehyde was consumed by pRCC and HK-2 cell lines but at a lower extent for Caki-2 and ACHN. 2-Pentadecanone, acetophenone and cyclohexanone exhibited a distinct profile in Caki-2 when compared with HK-2, ACHN and blanks, characterized by higher consumption by this cell line. Acetaldehyde revealed an increased consumption in both pRCC cell lines (Table 2).

### 2.2. Volatile Exometabolome Signature of Metastatic versus Non-Metastatic RCC Cell Lines and ccRCC versus pRCC Cell Lines

**Figure 4:**
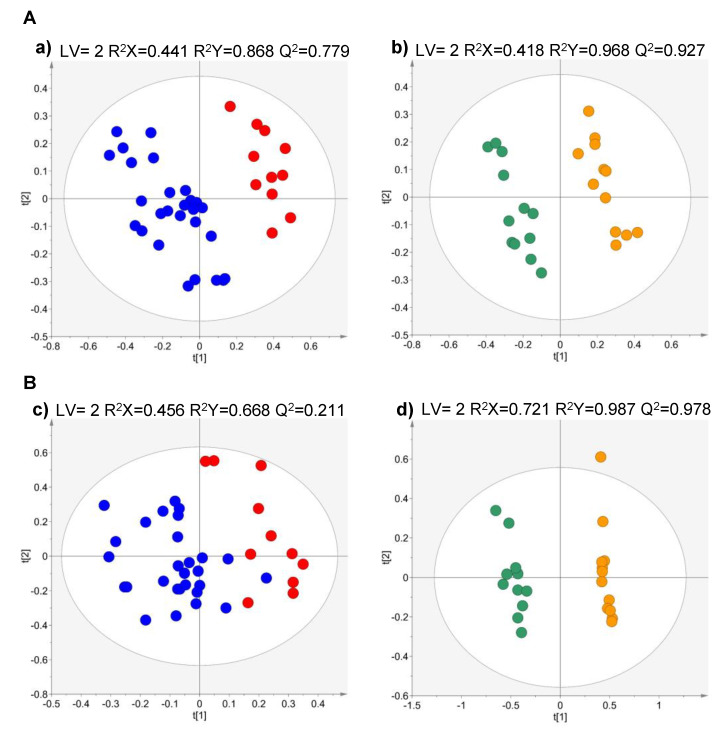
Partial least squares-discriminant analysis (PLS-DA) scores scatter plots obtained for the HS-SPME/GC–MS chromatogram data of (**A**) VOCs and (**B**) VCCs of culture media of the metastatic cell lines vs. non-metastatic cell lines of each RCC histological subtype. (**a**,**c**) metastatic ccRCC (Caki-1, red, *n* = 11) *vs.* non-metastatic ccRCC (769-P and 786-O, dark blue, *n* = 27) and (**b**,**d**) metastatic pRCC (ACHN, green, *n* = 12) vs. non-metastatic pRCC (Caki-2, orange, *n* = 12).

To investigate the volatile exometabolome signature of RCC cell lines with different metastatic potential, the same strategy was applied to compare the extracellular medium of metastatic and non-metastatic RCC cell lines (Figure 4). A good separation was obtained for metastatic vs. non-metastatic ccRCC cells in VOCs analysis (Q^2^ > 0.7; Figure 4a), whereas a poor separation (Q^2^ = 0.211) was observed in VCCs (Figure 4c). For pRCC, a good separation with high predictive power (Q^2^ > 0.9) was observed in both analytical methodologies (Figure 4b,d).

The volatile exometabolome signature of metastatic ccRCC cell lines unveiled statistically significant alterations in the levels of 15 compounds when compared with non-metastatic cell lines (Table 3). For pRCC, 17 compounds were found significantly altered between metastatic and non-metastatic cells. Interestingly, most of these alterations were specific of the RCC histological subtype with only a decrease in the levels of decane, dodecane and 4-methylbenzaldehyde found in common for ccRCC and pRCC. In general, metastatic ccRCC cell lines mostly showed dysregulations in the levels of alkanes and alkenes (decreased and increased) and benzene derivatives (decreased) when compared with non-metastatic cells, while metastatic pRCC cell lines mostly showed alterations in the levels of aldehydes (decreased) and ketones (decreased and increased).

Finally, the comparison of the volatile exometabolome between ccRCC and pRCC unveiled poor separation in the PLS-DA models of VOCs and VCCs analysis (Figure S3). In the PLS-DA model of VOCs (Figure S3A), the extracellular media of the Caki-1 cell line was wrongly classified in LV1 positive. Nonetheless, the model showed high predictive power and some statistically significant alterations between ccRCC and pRCC were disclosed, namely in the levels of 2,4-dimethyl-1-heptene, 3-carene, 4-methylbenzaldehyde, 4-methylheptane and an unknown compound (Un RT 8.40 *m/z* 69; Table 3).

## 3. Discussion

The profiling of volatile compounds present in the culture medium (exometabolome) unveiled important alterations associated with the excretion and consumption of nutrients by RCC cells lines when compared with a non-tumor cell line. In addition, different RCC histological subtypes (ccRCC and pRCC), as well as different stages of the disease (metastatic and non-metastatic) disclosed characteristic exometabolome signatures. These metabolic alterations may constitute a panel of potential biomarkers yet to be investigated in urine from RCC patients, compared with cancer-free individuals. Common to all cancer cell lines under study, we found increased levels of 2-ethylhexanol, tetradecane, formaldehyde, acetone and an unknown compound (Un RT 10.18 *m/z* 58), and decreased levels of acetaldehyde and cyclohexanone. These results suggest that different histological RCC subtypes have, at least in part, a common regulatory mechanism that affects the metabolic pathways in which these compounds are intermediates.

In a general way, RCC cell lines present high levels of hydrocarbons (alkanes and alkenes) in the extracellular medium. The main mechanism that affects the production of hydrocarbons is the oxidative stress, since the production of these volatile metabolites is mainly due to the induction of ROS-mediated polyunsaturated fatty acid peroxidation [30]. In fact, some studies have already shown elevated ROS levels in RCC [31,32] and have also provided evidence that lipid peroxidation is an important mechanism in renal carcinogenesis [33,34].

Another major change observed in this study was a significant increase of some alcohols (namely 2-ethylhexanol) in RCC cell lines when compared to the non-tumorigenic cell line. These results can be explained by a possible up-regulation of cytochrome P450 (CYP450) enzymes, which can hydroxylate several volatiles formed during ROS-mediated lipid peroxidation, leading to the production of the corresponding alcohols [30]. This theory is supported by some studies [33,35,36] whose results demonstrated that some CYP isoforms (such as CYP1B1, CYP1A1, CYP2C19, CYP2D6, CYP2E1, CYP3A4 or CYP3A5) are altered in RCC patients. Furthermore, 2-ethylhexanol has already been found increased in preoperative vs. postoperative urine samples from RCC patients [27], indicating that it may be considered a potential translatable biomarker for non-invasive measurement in urine. However, 2-ethylhexanol has also been reported as a potential urinary biomarker of lung [37] and prostate [38] cancers, which indicates a lack of specificity towards RCC. Nevertheless, this biomarker can be combined with others to define a robust panel of biomarkers for early diagnosis of RCC.

In addition, our metabolomic results also showed that most of the aldehydes (e.g., 4-methylbenzaldehyde, 3-methylbenzaldehyde and formaldehyde) were significantly increased in RCC cell lines. Increased levels of aldehydes may derive from different mechanisms including a direct origin in the reduction of hydroperoxides by CYP450 or from the conversion of some alcohols to their corresponding aldehydes mediated by alcohol dehydrogenases (ADHs) [30]. Since the amount of some lipids in cancer cells membranes is greater than in normal cells, it can be assumed that the increase in aldehyde levels in RCC cell lines may be linked to changes in the membrane lipid composition subsequent to increased oxidative stress [30,31,32]. Furthermore, it is also known that RCC cells have a significantly higher activity of total ADH and ADH class I that is exacerbated in patients with a more advanced stage [39,40], meaning that tumoral cells have an increased ability to produce aldehydes, which can intensify carcinogenesis. In agreement, a significant enhancement of aldehydes has been previously shown in urine from RCC patients compared to healthy controls [27].

Remarkably, acetaldehyde, another well-known aldehyde, appears significantly decreased in all RCC cell lines when compared to the non-tumorigenic cell line. This finding is opposite to what would be expected based on aforementioned higher activity of ADH in renal cancer cells. The ability of acetaldehyde to bind to DNA and cellular proteins to form adducts may explain the results verified in the exometabolome [40]. Moreover, acetaldehyde can lead to activation of proto-oncogenes, inactivation of tumor suppressor genes in replicating cells and inhibition of many important enzymes of DNA synthesis pathways, playing an important role in carcinogenesis [40]. Further studies are needed to explore whether RCC cells possess/induce detoxifying mechanisms for this toxic and reactive aldehyde.

Ketones were also found altered in the exometabolome of RCC cells compared with the non-tumor cell line, with special emphasis for acetone whose levels are significantly increased. Several studies [15,23,41] have associated this class of volatile compounds with altered pathways in cancer, namely the propensity of tumor cells to favor their metabolism via glycolysis (Warburg effect) with a consequent increase in lactate secretion. These will lead to an accumulation of acetyl-CoA, which in turn results in an increased production of ketone bodies, namely acetone [30].

In contrast, cyclohexanone was found decreased in the exometabolome of all RCC cells. Of note, this finding is not in agreement with the previous study by Wang et al. [27] where they showed significantly higher levels of cyclohexanone in preoperative urines from RCC patients. Although these contradictory results may be a result of sources of residual variation as diet, medication use, gender, age and comorbidities, among others, which may affect urine, further studies should be carried out in the future. Noticeably, this volatile compound is highly consumed by the pRCC cell line Caki-2 compared to other cancer cell lines. Caki-2 revealed a different profile since the levels of 2-pentadecanone and an unknown compound (Un RT 9.80, *m/z* 59) were found significantly altered only in this pRCC cell line. Contrary to other RCC histological subtypes, ccRCC and pRCC arise from adjacent parts of the proximal tubule [42], which may justify the poor discrimination found between both subtypes. Nevertheless, some volatile compounds were found to be discriminative between ccRCC and pRCC subtypes, mainly belonging to hydrocarbons, aldehydes and ketone classes. Although differences in the metabolic profile of ccRCC and pRCC subtypes have already been reported in terms of amino acid and fatty acid metabolism, whose levels were found to be increased in ccRCC [43], thus being associated with the higher aggressiveness of ccRCC comparing to the pRCC subtype [42,44], our study reports for the first time differences in the volatile exometabolome of these two RCC subtypes.

Regarding the comparison metastatic vs. non-metastatic stage in both the clear cell and the papillary RCC cell lines, the results suggest a variation of the volatile compounds in a stage-dependent manner since statistically significant differences in the levels of several metabolites were found. Curiously, the separation between metastatic and non-metastatic cell lines was more robust in the pRCC subtype, which justifies the markedly altered profile of the pRCC cell line, Caki-2. These results are consistent with the findings reported by Schaeffeler et al. [42], which demonstrated that ccRCC-derived metastases showed similar metabolite levels when compared with primary ccRCC. Our results revealed common alterations in the levels of several alkanes (decanal, decane and dodecane), one aldehyde (4-methylbenzaldehyde) and one alcohol (cyclohexanol) in all metastatic cell lines compared with the non-metastatic. Indeed, several ketones and aldehydes were only found altered in the pRCC metastatic cell line, ACHN. However, our results disclosed decreased levels of aldehydes in the metastatic cell lines, which may be used in amino acid synthesis, as proposed by some other authors [23].

This study demonstrates for the first time, to our knowledge, the potential of volatile compounds in discriminating RCC cells from nonmalignant cells, according to the histological subtype and tumor stage. However, the metabolic pathways that lead to VOCs and VCCs production are not yet clearly understood, hindering an accurate interpretation of the results. Nevertheless, our findings support the hypothesis that altered energy production in RCC cells enables them to survive under oxidative stress conditions and to migrate to other tissues and form metastases [45]. This study also reinforces that the investigation of the volatile signature of the exometabolome using cancer cell lines can provide candidate biomarkers with potential to be translatable for urine of RCC patients, which may ultimately lead to the development of volatile sensor-based approach for non-invasive diagnosis of RCC.

## 4. Materials and Methods

### 4.1. Chemicals

All chemicals were of analytical grade and dissolved in deionized water unless otherwise indicated. RPMI-1640 medium, sodium chloride (NaCl, ≥ 99.5%), *O*-(2,3,4,5,6-pentafluorobenzyl) hydroxylamine (PFBHA, ≥ 99%) and thymol (≥ 98.5%) were obtained from Sigma-Aldrich (St. Louis, Missouri, USA). The antibiotic mixture penicillin/streptomycin (10.000 U/mL/10.000 mg/mL), heat-inactivated fetal bovine serum (FBS) and trypsin-EDTA (0.25%) were purchased from GIBCO Invitrogen (Barcelona, Spain). Hydrochloric acid (HCl) and sodium hydrogencarbonate were obtained from Merck (Darmstadt, Germany).

### 4.2. Cell lines and Culture Conditions

Five human RCC immortalized cell lines (769-P, 786-O, ACHN, Caki-1 and Caki-2) and one non-tumorigenic renal cell line (HK-2) were purchased from the American Type Culture Collection (ATCC; Manassas, VA, USA) and their characteristics are summarized in Table S1. The selection of tumorigenic cell lines was carried out to allow the study of the most common RCC subtypes (ccRCC and pRCC) at different disease stages. Thus, 769-P and 786-O are recognized as primary ccRCC while Caki-1 is a metastatic ccRCC cell line [46]. Furthermore, and despite some controversies in histological classification, Caki-2 has been recognized as a primary pRCC cell line expressing papillary characteristics and ACHN is considered a metastatic pRCC cell line [46]. HK-2 is an immortalized proximal tubule cell line derived from normal human kidney [47]. All cell lines were grown in RPMI-1640 supplemented with 10% of FBS and 1% penicillin/streptomycin and incubated at 37 °C and 5% CO_2_. All cell lines were routinely tested for *Mycoplasma* spp. contamination (PCR Mycoplasma Detection Set, TaKaRa).

For each cell line, two different cell stock vials were grown in parallel and the experiments carried out up to 7 consecutive passages in plastic T25 culture flasks, after an adaptation stage of at least three passages for all cell lines. After 24 h, a confluence of 50%–60% was observed, the culture medium was discarded and replaced with 7 mL of fresh culture medium (which was prepared in enough amount to maintain the same composition in all collected flasks, thus reducing possible bias). The culture flasks were maintained in the incubator for another 48 h reaching near confluence, together with blanks (T25 culture flasks with the same culture medium but without cells). After 48 h, the extracellular medium from each culture flask with cells and blanks was collected and centrifuged (5 min, 2000*g*, 4 °C). Then, the supernatant was divided into two 2.5 mL aliquots and immediately stored at −80 °C until analysis. A pool of all samples and blanks, called quality control (QCs), was also prepared under the same collection and storage conditions.

### 4.3. Sample Preparation and Volatile Extraction by HS-SPME

Sample preparation was performed as described in previous studies published by our group [21,24,28,48]. Briefly, stored samples were first thawed slowly on ice to minimize the loss of volatiles. For VOCs analysis, 2 mL of each extracellular culture medium was transferred to a 10 mL glass vial with 30 μL of the internal standard (thymol, 2 mg/L) and 0.43 g of NaCl (salting out effect). Then, VOCs were extracted through HS-SPME using a divinylbenzene/carboxen/polydimethylsiloxane (DVB/CAR/PDMS) fiber with an incubation time of 11 min and extraction of 30 min at 44 °C, under continuous stirring (250 rpm) [21,24,48]. For VCCs analysis, 2 mL of each culture medium was placed into a 10 mL glass vial with 47 μL of the derivatizing agent (*O*-(2,3,4,5,6-pentafluorobenzyl) hydroxylamine, 40 g/L) and the compounds were extracted using a polydimethylsiloxane/divinylbenzene (PDMS/DVB) fiber with an incubation time of 6 min at 62 °C and extraction using the same temperature during 51 min, under continuous stirring (250 rpm) [28,48]. All samples were randomly injected, and the QCs were injected at the same conditions, on every 6 samples, to evaluate the reproducibility in both analyses.

### 4.4. GC–MS Analysis: Equipment and Conditions

VOCs detection was accomplished using a 436-GC system (Bruker Daltonics, Fremont, CA) coupled to a SCION Single Quadrupole (SQ) mass detector and a Bruker Daltonics MS workstation software (version 8.2). A fused silica capillary column Rxi-5Sil MS (30 m × 0.25 mm × 0.25 μm; RESTEK Corporation, U.S., Bellefonte, Pennsylvania) was used and helium C-60 (Gasin, Portugal) was the carrier gas (flow rate 1 mL/min). The oven temperature was fixed at 40 °C for 1 min, increasing to 250 °C (rate 5 °C/min), held for 5 min, followed by increasing to 300 °C (rate 5 °C/min) and held for 1 min. The MS detector was operated in electron impact mode (70 eV) at 260 °C, the transfer line temperature was 250 °C and the manifold temperature was 40 °C. Data acquisition was performed in full scan mode with a mass range between 40 and 350 *m/z* at a scan rate of 6 scans/s. VCCs were analyzed using a 436-GC system (Bruker Daltonics, Fremont, CA) coupled to a EVOQ Triple Quadrupole (TQ) mass detector and a Bruker MS workstation software version 8.2. The column, carrier gas and oven temperature conditions were the same described above for VOCs analysis. The MS detector was operated in the electron impact mode (70 eV) at 270 °C, the temperature of transfer line and manifold were 260 °C and 40 °C, respectively. Data acquisition was performed in the full scan mode considering a 50–600 *m/z* mass range.

### 4.5. Compound Identification and GC–MS Data Pre-Processing

Compounds detected in both VOCs and VCCs analytical methodologies were identified by comparison of the MS fragmentation with the mass spectra present in the National Institute of Standards and Technology (NIST 14) database and by comparing the experimental Kovats retention index (RI) with literature. When possible, the identification was confirmed by comparison of the retention time and MS spectra of the samples with the commercially available standard compounds analyzed using the same conditions.

Before statistical analysis, the data obtained from GC–MS were converted into a manageable format (netcdf) and preprocessed using MZmine 2.53 [49]. The preprocessing steps consisted of filtering, peak detection, chromatogram deconvolution and alignment. The parameters used for preprocessing of VOCs profile were: RT range 2–32 minutes; *m/z* range 50–250; MS data noise level 1 × 10^4^; *m/z* tolerance 0.3; chromatogram baseline level 5 × 10^4^ and peak duration range 0.05–1 min. For VCCs analysis, the parameters used were RT range 9–45 minutes; *m/z* range 50–500; MS data noise level 1 × 10^4^; *m/z* tolerance 0.3; chromatogram baseline level 1 × 10^4^; peak duration range 0.03–0.2 min. GC contaminants from the chromatographic column or fiber (among others) were manually removed from the final data matrix.

### 4.6. Statistical Analysis

The statistical analysis pipeline used for VOCs and VCCs final matrix was the same. First, data were normalized by the total area (TA) of the chromatogram and scaled to Pareto. Then, PCA (unsupervised analysis) was applied to detect trends, outliers and to evaluate the distribution of the QCs, followed by PLS-DA (supervised analysis) to discriminate classes and to identify the specific metabolic signature of each cell line, in SIMCA 15.0.2 (Umetrics Umea, Sweden). A default sevenfold internal cross-validation and permutation testing were used to confirm the robustness of PLS-DA models. All volatile compounds with VIP (variable importance in the projection) higher than one were considered important for group discrimination and were subsequently submitted to the Mann–Whitney test. The false discovery rate (FDR) method was used to adjust *p*-values for multiple comparison using the MetaboAnalyst 4.0 [50], and compounds with *p*-value < 0.05 were considered statistically significant. In addition, the AUC (area under the curve) was computed for each discriminant compound using the same analytical platform. The effect size and the standard error were also computed as described by Berben et al. [29]. Finally, the average normalized area of each statistically significant compound in each sample group was represented in a heatmap in MetaboAnalyst 4.0.

## 5. Conclusions

In this study, we used an in vitro metabolomics approach to obtain the volatile signatures of RCC cells with distinct histological subtypes and stages. The results showed that alterations in the volatile profile are capable of discriminating RCC from non-tumorigenic renal cells. This unique volatile profile of RCC cells can be useful to unveil promising noninvasive biomarkers for early diagnosis in more complex biological matrices, such as urine. A multi-biomarker panel can provide a more robust and accurate tool for RCC diagnosis than the use of only one metabolite as a biomarker. Indeed, 2-ethylhexanol, tetradecane, formaldehyde, acetone, cyclohexanone and acetaldehyde were able to discriminate RCC cell lines from the non-tumor cell line and may constitute a potential panel of candidate biomarkers for RCC diagnosis. This study also demonstrated that the volatile profile of metastatic cell lines markedly differed from the non-metastatic in each RCC subtype, indicating some promising biomarkers applicable to the categorization of tumor stage, pending further investigation and validation.

## Figures and Tables

**Table 1:** List of volatile compounds (VOCs and VCCs) significantly altered between each clear cell renal cell carcinoma (ccRCC; 769-P, 786-O and Caki-1) cell line and the non-tumoral cell line (HK-2).

Metabolite	769-P (*n* = 13) vs. HK-2 (*n* = 12)				786-O (*n* = 14) vs. HK-2 (*n* = 12)				Caki-1 (*n* = 11) vs. HK-2 (*n* = 12)		
	ES ± SE	*p*-Value ^a^	AUC		ES ± SE	*p*-Value ^a^	AUC		ES ± SE	*p*-Value ^a^	AUC
***Alcohols***											
2-Ethylhexanol ^b, L2,^ *	↑1.42 ± 0.86 ^E^	1.26 × 10^−3^	0.885		↑0.94 ± 0.79 ^E^	4.88 × 10^−2^	0.738		↑2.39 ± 1.05 ^E^	6.21 × 10^−4^	0.955
2,6-Dimethyl-7-octen-2-ol *^b, L2^*									↓−1.79 ± 0.94 ^C^	2.02 × 10^−3^	0.916
Cyclohexanol *^b, L2^*					↓−1.29 ± 0.82 ^C^	8.06 × 10^−3^	0.821				
Levomenthol *^b, L2^*	↓−0.84 ± 0.79	4.90 × 10^−2^	0.734						↓−1.30 ± 0.87 ^C^	2.96 × 10^−2^	0.818
***Alkanes***											
2-Ethoxy-2-methyl-propane *^b, L2^*					↑1.01 ± 0.80 ^C^	3.00 × 10^−2^	0.762				
Decane *^b, L2^*	↑0.84 ± 0.79	4.90 ×10^−2^	0.734		↑1.12 ± 0.81 ^E^	2.23 × 10^−2^	0.780				
Tetradecane ^c, L2,^ *	↑3.68 ± 1.25 ^E^	2.90 × 10^−7^	1.000		↑2.81 ± 1.07 ^E^	2.90 × 10^−7^	1.000		↑1.88 ± 0.94 ^E^	5.61 × 10^−4^	0.909
Dodecane *^b, L2^*									↓−1.29 ± 0.87 ^C^	2.96 × 10^−2^	0.811
***Alkenes***											
2,4-Dimethyl-1-heptene *^b, L2^*	↑0.80 ± 0.79 ^C^	3.00 × 10^−2^	0.769		↑0.89 ± 0.78 ^C^	2.23 × 10^−2^	0.780				
3-Carene *^b, L1^*	↑2.32 ± 0.99	4.33 × 10^−5^	0.962		↑1.84 ± 0.90	2.59 × 10^−4^	0.940				
***Aldehydes***											
4-Methylbenzaldehyde *^b, L2^*	↑2.00 ± 0.94 ^C^	1.60 × 10^−4^	0.936		↑1.69 ± 0.88 ^C^	1.10 × 10^−3^	0.899				
Benzaldehyde *^b, L1^*					↓−1.59 ± 0.86 ^C^	2.74 × 10^−3^	0.863				
3-Methylbenzaldehyde *^c, L2^*	↑1.75 ± 0.89 ^E^	2.34 × 10^−5^	0.946		↑2.03 ± 0.93 ^E^	9.32 × 10^−6^	0.959				
Acetaldehyde *^c, L1^*	↓−10.93 ± 3.06 ^C^	2.90 × 10^−7^	1.000		↓−4.48 ± 1.43 ^C^	2.90 × 10^−7^	1.000		↓−4.77 ± 1.55 ^C^	5.18 × 10^−6^	0.992
Formaldehyde *^c, L2^*	↑3.34 ± 1.18 ^C^	2.90 × 10^−7^	1.000		↑2.58 ± 1.02 ^C^	2.90 × 10^−7^	1.000		↑3.13 ± 1.18 ^C^	5.18 × 10^−6^	1.000
Decanal ^b, L1,^ *	↓−1.72 ± 0.90 ^C^	1.61 × 10^−3^	0.872		↓−1.87 ± 0.90 ^C^	3.00 × 10^−3^	0.857				
***Benzene Derivatives***											
Ethylbenzene *^b, L2^*	↑1.46 ± 0.86	3.58 × 10^−4^	0.917		↑1.00 ± 0.79 ^E^	2.42 × 10^−3^	0.875				
Styrene ^b, L2,^ *	↑1.32 ± 0.84 ^E^	1.61 × 10^−3^	0.871								
Xylene *^b, L2^*	↑1.61 ± 0.88 ^E^	4.33 × 10^−5^	0.962		↑1.11 ± 0.81 ^E^	6.27 × 10^−4^	0.917				
***Ketones***											
Acetophenone *^b, L2^*	↓− 0.96 ± 0.80^C^	4.31 × 10^−2^	0.750								
Cyclohexanone *^b, L2,^* *	↓− 3.29 ± 1.19 ^C^	2.88 × 10^−6^	1.000		↓−1.53 ± 0.85 ^C^	2.74 × 10^−3^	0.863		↓−1.54 ± 0.91 ^C^	6.04 × 10^−3^	0.879
Acetone *^c, L2^*	↑2.12 ± 0.94 ^E^	5.63 × 10^−5^	0.929		↑2.22 ± 0.96 ^E^	7.25 × 10^−6^	0.964		↑1.33 ± 0.86 ^E^	6.56 × 10^−3^	0.833
***Unknowns***											
Un (RT 8.40, *m/z* 69) *^b, L4^*	↑1.44 ± 0.86 ^C^	1.43 × 10^−2^	0.801		↑1.53 ± 0.85 ^C^	3.31 × 10^−3^	0.851				
Un (RT 10.18, *m/z* 58) ^c, *L4*^	↑3.64 ± 1.24 ^E^	2.90 × 10^−7^	1.000		↑3.07 ± 1.12 ^E^	2.90 × 10^−7^	1.000		↑2.04 ± 0.97 ^E^	5.18 × 10^−6^	0.992
Un (RT 12.82. *m/z* 69) *^b, L4^*					↑0.98 ± 0.79	2.40 × 10^−2^	0.774				
Un (RT 16.64, *m/z* 61) *^b, L4^*	↓− 4.16 ± 1.38	2.88 × 10^−6^	1.000		↓−4.39 ± 1.41 ^E^	3.73 × 10^−6^	1.000				

ES: Effect size, SE: standard error, AUC: area under the curve. ES and SE were determined as described in reference Berben et al. (2012) [29]. ^a^ False discovery rate adjusted *p*-value; *^b, c^* Compounds detected through VOCs and VCCs analytical methods, respectively. ↑ and ↓: volatile compound found increased and decreased, respectively, in the extracellular medium of RCC compared with the non-tumoral cell line. ^E, C^ Metabolites excreted and consumed, respectively, when compared with blanks. No indication was added when the levels of the metabolites in tumoral cell lines were similar to that of blanks. *^L1^* Identified metabolites (GC–MS analysis of the metabolite of interest and a chemical reference standard under identical analytical conditions within the same laboratory).*^L2^* Putatively annotated compounds (spectral MS similarity with the NIST database).*^L4^* Unknown compounds. * Metabolites previously reported as potential biomarkers of RCC in urine [27].

**Table 2:** List of volatile compounds (VOCs and VCCs) significantly altered between papillary renal cell carcinoma (pRCC; Caki-2 and ACHN) cell line and the non-tumoral cell line (HK-2).

Metabolite	Caki-2 (*n* = 12) vs. HK-2 (*n* = 12)				ACHN (*n* = 12) vs. HK-2 (*n* = 12)		
	ES ± SE	*p*-Value ^a^	AUC		ES ± SE	*p*-Value ^a^	AUC
***Alcohols***							
2-Ethylhexanol ^b, L2,^ *	↑2.51 ± 1.05 ^E^	2.22 × 10^−5^	0.972		↑4.29 ± 1.44 ^E^	7.40 × 10^−6^	1.000
Cyclohexanol *^b, L2^*	↑1.45 ± 0.87 ^E^	2.87 × 10^−3^	0.868				
***Alkanes***							
Tetradecane ^c, L2,^ *	↑3.15 ± 1.18 ^E^	8.63 × 10^−7^	1.000		↑2.50 ± 1.05 ^E^	1.73 × 10^−6^	1.000
***Aldehydes***							
4-Methylbenzaldehyde *^b, L2^*					↓−1.84 ± 0.93 ^C^	4.78 × 10^−3^	0.875
3-Methylbenzaldehyde *^c, L2^*	↑0.81 ± 0.81 ^C^	1.21 × 10^−2^	0.799				
Acetaldehyde *^c, L1^*	↓−9.59 ± 2.82 ^C^	8.63 × 10^−7^	1.000		↓−10.95 ± 3.19 ^C^	1.73 × 10^−6^	1.000
Formaldehyde *^c, L2^*	↑4.39 ± 1.46^C^	8.63 × 10^−7^	1.000		↑2.34 ± 1.02 ^C^	7.25 × 10^−6^	0.979
***Ketones***							
2-Pentadecanone *^b, L1^*	↓−2.64 ± 1.07	9.86 × 10^−6^	0.986				
Acetophenone *^b, L2^*	↓−4.85 ± 1.57 ^C^	3.70 × 10^−6^	1.000				
Cyclohexanone ^b, L2,^ *	↓−7.09 ± 2.15 ^C^	3.70 × 10^−6^	1.000		↓−1.40 ± 0.87 ^C^	4.78 × 10^−3^	0.868
Acetone *^c, L2^*	↑4.63 ± 1.52 ^E^	8.63 × 10^−6^	1.000		↑2.89 ± 1.13 ^E^	1.04 × 10^−5^	0.972
***Unknowns***							
Un (RT 9.80, *m/z* 59) *^b, L4^*	↑1.55 ± 0.89 ^C^	3.05 × 10^−3^	0.861				
Un (RT 10.18, *m/z* 58) ^c, *L4*^	↑4.65 ± 1.52 ^E^	8.63 × 10^−7^	1.000		↑3.38 ± 1.23 ^E^	1.73 × 10^−6^	1.000
Un (RT 12.82. *m/z* 69) *^b, L4^*	↓−0.99 ± 0.82 ^C^	2.47 × 10^−6^	0.785				
Un (RT 16.64, *m/z* 61) *^b, L4^*					↓−1.23 ± 0.85 ^E^	1.13 × 10^−2^	0.833

ES: effect size, SE: standard error, AUC: area under the curve. ES and SE were determined as described in reference Berben et al. (2012) [29]. ^a^ False discovery rate adjusted *p*-value; *^b, c^* Compounds detected through VOCs and VCCs analytical methods, respectively. ↑ and ↓: volatile compound found increased and decreased, respectively, in the extracellular medium of RCC compared with non-tumoral cell line. ^E, C^ Metabolites excreted and consumed, respectively, when compared with blanks. No indication was added when the levels of the metabolites in tumoral cell lines were similar to that of blanks. *^L1^* Identified metabolites (GC–MS analysis of the metabolite of interest and a chemical reference standard under identical analytical conditions within the same laboratory). *^L2^* Putatively annotated compounds (spectral MS similarity with the NIST database). *^L4^* Unknown compounds. * Metabolites previously reported as potential biomarkers of RCC in urine [27].

**Table 3:** List of volatile compounds (VOCs and VCCs) significantly altered between metastatic cell lines compared with non-metastatic cell lines and between RCC histological subtype (ccRCC and pRCC).

Metabolite	Metastatic ccRCC (*n* = 11) vs. Non-Metastatic ccRCC (*n* = 27)				Metastatic pRCC (*n* = 12) vs. Non-Metastatic pRCC (*n* = 12)				ccRCC (*n* = 38) vs. pRCC (*n* = 24)		
	ES ± SE	*p*-Value ^a^	AUC		ES ± SE	*p*-Value ^a^	AUC		ES ± SE	*p*-Value ^a^	AUC
***Alcohols***											
2-Ethylhexanol *^b, L2^*					↑1.20 ± 0.84 ^E^	1.19 × 10^−2^	0.828		↓−0.80 ± 0.52 ^E^	1.88 × 10^−3^	0.730
Cyclohexanol *^b, L2^*	↑1.83 ± 0.80 ^E^	1.71 × 10^−4^	0.882		↓−2.61 ± 1.07	7.40 × 10^−6^	0.993		↑1.15 ± 0.54 ^C^	2.04 × 10^−5^	0.813
***Alkanes***											
2-Ethoxy-2-methyl-propane *^b, L2^*	↓−0.99 ± 0.72 ^C^	7.42 × 10^−3^	0.781						↑0.95 ± 0.53	1.13 × 10^−4^	0.763
Decane *^b, L2^*	↓−2.54 ± 0.89 ^C^	1.05 × 10^−7^	0.989		↓−0.96 ± 0.82 ^C^	1.67 × 10^−2^	0.806		↓−0.98 ± 0.53	2.89 × 10^−4^	0.728
Dodecane *^b, L2^*	↓−2.33 ± 0.86	1.35 × 10^−6^	0.959		↓−1.28 ± 0.85	1.81 × 10^−2^	0.799		↑1.03 ± 0.54	9.68 × 10^−5^	0.782
4-Methylheptane *^b, L2^*	↓−1.40 ± 0.75 ^C^	2.60 × 10^−4^	0.872						↑1.28 ± 0.55 ^C^	1.31 × 10^−6^	0.839
Tetradecane *^c, L2^*					↓−1.64 ± 0.90 ^E^	5.78 × 10^−4^	0.896				
***Alkenes***											
2,4-Dimethyl-1-heptene *^b, L2^*	↓−1.60 ± 0.77 ^C^	5.02 × 10^−5^	0.906						↑1.29 ± 0.55 ^C^	1.31 × 10^−6^	0.842
3-Carene *^b, L1^*	↓−2.29 ± 0.86 ^C^	2.99 × 10^−8^	1.000						↑1.36 ± 0.56	6.99 × 10^−7^	0.850
***Aldehydes***											
4-Methylbenzaldehyde *^b, L2^*	↓−2.26 ±0.85 ^C^	1.89 × 10^−7^	0.983		↓−1.23 ± 0.85 ^C^	2.79 × 10^−2^	0.778		↑1.28 ± 0.55 ^C^	2.22 × 10^−6^	0.827
3-Methylbenzaldehyde *^c, L2^*					↓−0.97 ± 0.82 ^C^	3.84 × 10^−4^	0.910				
Acetaldehyde *^c, L1^*					↓−1.90 ± 0.94 ^C^	3.84 × 10^−4^	0.917				
Formaldehyde *^c, L2^*					↓−1.29 ± 0.85 ^C^	5.56 × 10^−3^	0.826				
Decanal *^b, L1^*	↑2.58 ± 0.90 ^E^	7.25 × 10^−7^	0.969		↓−1.07 ± 0.83 ^E^	1.13 ×10^−2^	0.833		↑0.54 ± 0.51 ^E^	3.29 × 10^−2^	0.654
***Benzene Derivatives***											
Styrene *^b, L2^*	↓−1.46 ± 0.76	6.20 × 10^−4^	0.852								
Xylene *^b, L2^*	↓−0.67 ± 0.70 ^E^	3.38 × 10^−3^	0.808								
Ethylbenzene *^b, L2^*	↓−0.51± 0.70 ^C^	7.12 × 10^−3^	0.784								
***Ketones***											
2-Pentadecanone *^b, L1^*					↑2.80 ± 1.11 ^E^	1.11 × 10^−5^	0.986		↑0.84 ± 0.53 ^E^	8.89 × 10^−4^	0.734
4-Methyl-2-hexanone *^b, L2^*									↑0.97 ± 0.53	9.68 × 10^−5^	0.768
Acetone *^c, L2^*					↓−1.97 ± 0.95 ^E^	3.84 × 10^−4^	0.910				
Acetophenone *^b, L2^*					↑5.83 ± 1.82 ^C^	5.55 × 10^−6^	1.000		↓−1.40 ± 0.56 ^C^	2.03 × 10^−4^	0.761
Cyclohexanone *^b, L2^*					↑8.14 ± 2.44 ^C^	7.40 × 10^−6^	1.000				
***Unknowns***											
Un (RT 8.40, *m/z* 69) *^b, L4^*	↓−2.02 ± 0.82 ^C^	2.86 × 10^−6^	0.949						↓−1.31 ± 0.55 ^E^	6.89 × 10^−6^	0.795
Un (RT 9.45 m/z 71) *^b, L4^*					↑1.79 ± 0.92	1.56 × 10^−2^	0.813				
Un (RT 9.80, *m/z* 59) *^b, L4^*					↓−1.79 ± 0.92	4.33 × 10^−4^	0.924				
Un (RT 10.18, *m/z* 58) ^c, *L4*^					↓−1.69 ± 0.91 ^E^	3.84 × 10^−4^	0.917				
Un (RT 12.82. *m/z* 69) *^b, L4^*	↑1.70 ± 0.79 ^C^	2.00 × 10^−5^	0.918								
Un (RT 16.64, *m/z* 61) *^b, L4^*	↑3.58 ± 1.06 ^E^	8.00 × 10^−7^	0.966						↑1.40 ± 0.56 ^C^	6.99 × 10^−7^	0.844

ES: effect size, SE: standard error, AUC: area under the curve. ES and SE determined as described in reference Berben et al. (2012) [29]. ^a^ False discovery rate adjusted *p*-value; *^b, c^* Compounds detected through VOCs and VCCs analytical methods, respectively. ↑ and ↓: Volatile compound found increased and decreased, respectively, in the extracellular medium of metastatic cell lines compared with non-metastatic cell lines and between RCC histological subtype (ccRCC and pRCC). ^E, C^ Metabolites excreted and consumed, respectively, when compared with blanks. No indication was added when the levels of the metabolites in tumoral cell lines were similar to that of blanks. *^L1^* Identified metabolites (GC–MS analysis of the metabolite of interest and a chemical reference standard under identical analytical conditions within the same laboratory). *^L2^* Putatively annotated compounds (spectral MS similarity with the NIST database). *^L4^* Unknown compounds.

## Supplementary Materials

The following are available online at https://www.mdpi.com/2218-1989/10/5/174/s1, Table S1: Characteristics of RCC cell lines (769-P, 786-O, Caki-1, Caki-2 and ACHN) and non-tumoral cell line (HK-2); Figure S1: HS-SPME-GC–MS chromatograms of (A) VOCs and (B) VCCs of QC culture medium. 1: 2-ethyl-hexanol; 2: acetophenone; 3: dodecane; 4: decanal; 5: dodecanal; 6: 2-pentadecanone; 7: formaldehyde; 8: acetaldehyde; 9: acetone; 10: tetradecane; 11: 2-octanone; 12: 3-methyl-benzaldehyde; Figure S2: PCA scores scatter plots obtained for the HS-SPME/GC–MS chromatogram data of (A) VOCs and (B) VCCs of culture media of QCs (dark blue, *n* = 15) vs. all cell lines and blanks (green, *n* = 88); Figure S3: PLS-DA scores scatter plots obtained for the HS-SPME/GC–MS chromatogram data of (A) VOCs and (B) VCCs of culture media of ccRCC cell lines (769-P, 786-O and Caki-1, light blue, *n* = 38) vs. pRCC cell lines (Caki-2 and ACHN, red, *n* = 24); In vitro RCC data.xlsx file containing all data obtained in this study.
