# Supplementary material for: Volatilomics Reveals Potential Biomarkers for Identification of Renal Cell Carcinoma: An In Vitro Approach
Source: Metabolites. 2020 Apr 27;10(5):174. doi: 10.3390/metabo10050174 (PMC7281256; doi:10.3390/metabo10050174)
Supplement: Supplementary file 1 [file metabolites-10-00174-s001.zip › Supplementary Material/Supplementary material.docx]

**Table S1:** Characteristics of RCC cell lines (769-P, 786-O, Caki-1, Caki-2 and ACHN) and non-tumoral cell line (HK-2).

|  | HK-2 | 769-P | 786-O | Caki-1 | Caki-2 | ACHN |
| --- | --- | --- | --- | --- | --- | --- |
| Organism | *Homo sapiens* | *Homo sapiens* | *Homo sapiens* | *Homo sapiens* | *Homo sapiens* | *Homo sapiens* |
| Age / years | Adult | 63 | 58 | 49 | 69 | 22 |
| Ethnicity | NA | Caucasian | Caucasian | Caucasian | Caucasian | Caucasian |
| Gender | Male | Female | Male | Male | Male | Male |
| Tissue | Kidney, cortex/ proximal tube | Kidney | Kidney | Kidney, derived from metastatic site: skin | Kidney | Kidney, derived from metastatic site: pleural effusion |
| Morphology | Epithelial | Epithelial | Epithelial | Epithelial | Epithelial | Epithelial |
| Culture Properties | Adherent | Adherent | Adherent | Adherent | Adherent | Adherent |
| Disease | Papilloma | Primary cell carcinoma | Primary cell carcinoma | Clear cell carcinoma | Clear cell carcinoma/ papillary | Renal cell adenocarcinoma |
| Tumorigenic | No | Yes | Yes | Yes | Yes | Yes |
| Metastatic Potential | - | No | No | Yes | No | Yes |

*NA: not available*

**Figure S1:** HS-SPME-GC-MS chromatograms of (A) VOCs and (B) VCCs of QC culture medium**.** 1: 2-ethyl-hexanol; 2: acetophenone; 3: dodecane; 4: decanal; 5: dodecanal; 6: 2-pentadecanone; 7: formaldehyde; 8: acetaldehyde; 9: acetone; 10: tetradecane; 11: 2-octanone; 12: 3-methyl-benzaldehyde.


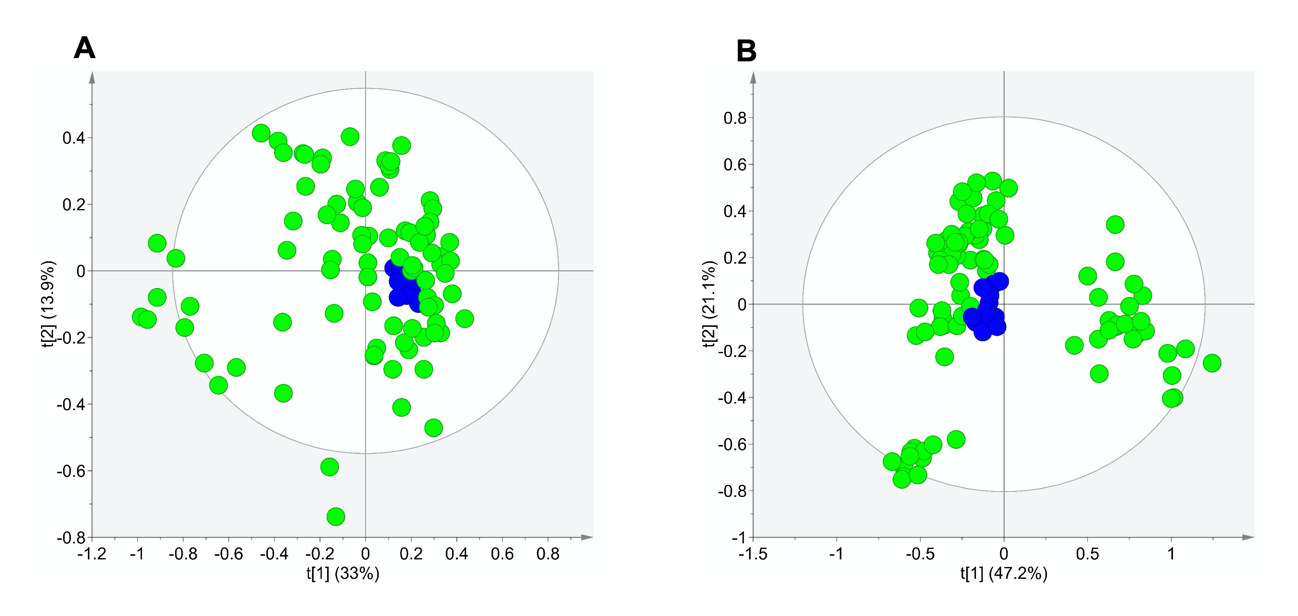
**Figure S2:** PCA scores scatter plots obtained for the HS-SPME/GC-MS chromatogram data of (A) VOCs and (B) VCCs of culture media of QCs (dark blue, n=15) vs. all cell lines and blanks (green, n=88).

**
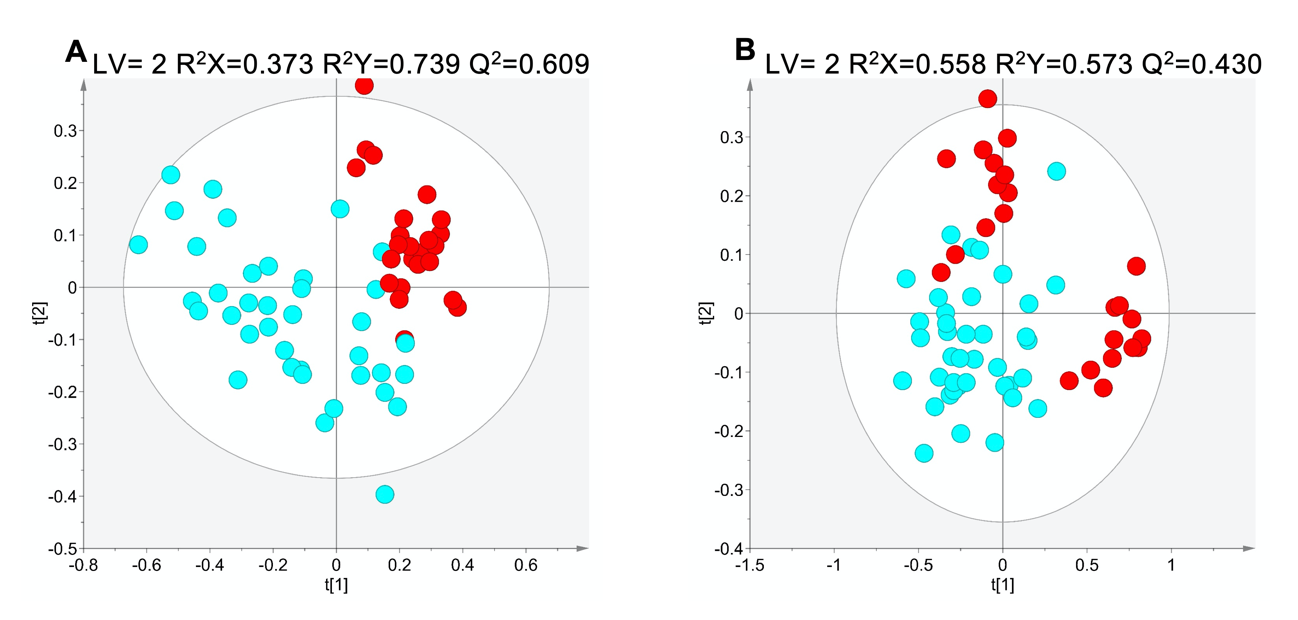
Figure S3:** PLS-DA scores scatter plot obtained for the HS-SPME/GC-MS chromatogram data of (A) VOCs and (B) VCCs of culture media of ccRCC cell lines (769-P, 786-O and Caki-1, light blue, n=38) vs. pRCC cell lines (Caki-2 and ACHN, red, n=24).
